# Supplementary material for: HIVprotI: an integrated web based platform for prediction and design of HIV proteins inhibitors
Source: J Cheminform. 2018 Mar 9;10:12. doi: 10.1186/s13321-018-0266-y (PMC5845081; doi:10.1186/s13321-018-0266-y)
Supplement: Supplementary file 2 — Additional file 2. Source code of HIVProtI web server. [file 13321_2018_266_MOESM2_ESM.zip › HIVprotI_Source-code/hivprotI/weka-3-6-6/doc/weka/classifiers/functions/supportVector/RegOptimizer.html]

RegOptimizer


---


|  |  |  |
| --- | --- | --- |
| **Overview** \| **Package** \| **Class** \| **Tree** \| **Deprecated** \| **Index** \| **Help** \| **Weka's home** | |  |
| **PREV CLASS**   **NEXT CLASS** | **FRAMES**    **NO FRAMES**     **All Classes** |  |
| SUMMARY: NESTED \| FIELD \| CONSTR \| METHOD | DETAIL: FIELD \| CONSTR \| METHOD |  |


---


## weka.classifiers.functions.supportVector Class RegOptimizer

```
java.lang.Object
  weka.classifiers.functions.supportVector.RegOptimizer
```

**All Implemented Interfaces:**: java.io.Serializable, OptionHandler, RevisionHandler

**Direct Known Subclasses:**: RegSMO

---

``` public class RegOptimizer extends java.lang.Object implements OptionHandler, java.io.Serializable, RevisionHandler ```

Base class implementation for learning algorithm of SMOreg
Valid options are:

```
 -L <double>
  The epsilon parameter in epsilon-insensitive loss function.
  (default 1.0e-3)
```

```
 -W <double>
  The random number seed.
  (default 1)
```

**Version:**
:   $Revision: 6622 $

**Author:**
:   Remco Bouckaert (remco@cs.waikato.ac.nz,rrb@xm.co.nz)

**See Also:**: Serialized Form

---

| **Field Summary** | |
| --- | --- |
| `double[]` | `m_alpha`             alpha and alpha\* arrays containing weights for solving dual problem |
| `double[]` | `m_alphaStar` |


**Constructor Summary**

`RegOptimizer()`
  
          the default constructor


| **Method Summary** | |
| --- | --- |
| `void` | `buildClassifier(Instances data)`             learn SVM parameters from data. |
| `java.lang.String` | `epsilonParameterTipText()`             Returns the tip text for this property |
| `int` | `getCacheHits()`             return the number of kernel cache hits |
| `double` | `getEpsilonParameter()`             Get the value of epsilon parameter of the epsilon insensitive loss function. |
| `int` | `getKernelEvaluations()`             returns the number of kernel evaluations |
| `java.lang.String[]` | `getOptions()`             Gets the current settings of the classifier. |
| `java.lang.String` | `getRevision()`             Returns the revision string. |
| `int` | `getSeed()`             Gets the current seed value for the random number generator |
| `java.util.Enumeration` | `listOptions()`             Gets an enumeration describing the available options. |
| `boolean` | `modelBuilt()`             flag to indicate whether the model was built yet |
| `java.lang.String` | `seedTipText()`             Returns the tip text for this property |
| `void` | `setEpsilonParameter(double v)`             Set the value of epsilon parameter of the epsilon insensitive loss function. |
| `void` | `setOptions(java.lang.String[] options)`             Parses a given list of options. |
| `void` | `setSeed(int value)`             Sets the seed value for the random number generator |
| `void` | `setSMOReg(SMOreg value)`             sets the parent SVM |
| `double` | `SVMOutput(Instance inst)` |
| `java.lang.String` | `toString()`             Prints out the classifier. |

**Methods inherited from class java.lang.Object**

`equals, getClass, hashCode, notify, notifyAll, wait, wait, wait`

**Field Detail**

### m\_alpha

```
public double[] m_alpha
```

:   alpha and alpha\* arrays containing weights for solving dual problem

---


### m\_alphaStar

```
public double[] m_alphaStar
```


**Constructor Detail**

### RegOptimizer

```
public RegOptimizer()
```

:   the default constructor


**Method Detail**

### listOptions

```
public java.util.Enumeration listOptions()
```

:   Gets an enumeration describing the available options.

    :   **Specified by:**: `listOptions` in interface `OptionHandler`
    :   **Returns:**: an enumeration of all the available options.

---


### setOptions

```
public void setOptions(java.lang.String[] options)
                throws java.lang.Exception
```

:   Parses a given list of options.


    Valid options are:

    ```
     -L <double>
      The epsilon parameter in epsilon-insensitive loss function.
      (default 1.0e-3)
    ```

    ```
     -W <double>
      The random number seed.
      (default 1)
    ```

    :   **Specified by:**: `setOptions` in interface `OptionHandler`
    :   **Parameters:**: `options` - the list of options as an array of strings **Throws:**: `java.lang.Exception` - if an option is not supported

---


### getOptions

```
public java.lang.String[] getOptions()
```

:   Gets the current settings of the classifier.

    :   **Specified by:**: `getOptions` in interface `OptionHandler`
    :   **Returns:**: an array of strings suitable for passing to setOptions

---


### modelBuilt

```
public boolean modelBuilt()
```

:   flag to indicate whether the model was built yet

    :   **Returns:**: true if the model was built

---


### setSMOReg

```
public void setSMOReg(SMOreg value)
```

:   sets the parent SVM

    :   **Parameters:**: `value` - the parent SVM

---


### getKernelEvaluations

```
public int getKernelEvaluations()
```

:   returns the number of kernel evaluations

    :   **Returns:**: the number of kernel evaluations

---


### getCacheHits

```
public int getCacheHits()
```

:   return the number of kernel cache hits

    :   **Returns:**: the number of hits

---


### buildClassifier

```
public void buildClassifier(Instances data)
                     throws java.lang.Exception
```

:   learn SVM parameters from data.
    Subclasses should implement something more interesting.

    :   **Parameters:**: `data` - the data to work with **Throws:**: `java.lang.Exception` - always an Exceoption since subclasses must override it

---


### SVMOutput

```
public double SVMOutput(Instance inst)
                 throws java.lang.Exception
```

:   **Parameters:**: `inst` - **Returns:**: **Throws:**: `java.lang.Exception`

---


### seedTipText

```
public java.lang.String seedTipText()
```

:   Returns the tip text for this property

    :   **Returns:**: tip text for this property suitable for displaying in the explorer/experimenter gui

---


### getSeed

```
public int getSeed()
```

:   Gets the current seed value for the random number generator

    :   **Returns:**: the seed value

---


### setSeed

```
public void setSeed(int value)
```

:   Sets the seed value for the random number generator

    :   **Parameters:**: `value` - the seed value

---


### epsilonParameterTipText

```
public java.lang.String epsilonParameterTipText()
```

:   Returns the tip text for this property

    :   **Returns:**: tip text for this property suitable for displaying in the explorer/experimenter gui

---


### getEpsilonParameter

```
public double getEpsilonParameter()
```

:   Get the value of epsilon parameter of the epsilon insensitive loss function.

    :   **Returns:**: Value of epsilon parameter.

---


### setEpsilonParameter

```
public void setEpsilonParameter(double v)
```

:   Set the value of epsilon parameter of the epsilon insensitive loss function.

    :   **Parameters:**: `v` - Value to assign to epsilon parameter.

---


### toString

```
public java.lang.String toString()
```

:   Prints out the classifier.

    :   **Overrides:**: `toString` in class `java.lang.Object`
    :   **Returns:**: a description of the classifier as a string

---


### getRevision

```
public java.lang.String getRevision()
```

:   Returns the revision string.

    :   **Specified by:**: `getRevision` in interface `RevisionHandler`
    :   **Returns:**: the revision


---


|  |  |  |
| --- | --- | --- |
| **Overview** \| **Package** \| **Class** \| **Tree** \| **Deprecated** \| **Index** \| **Help** \| **Weka's home** | |  |
| **PREV CLASS**   **NEXT CLASS** | **FRAMES**    **NO FRAMES**     **All Classes** |  |
| SUMMARY: NESTED \| FIELD \| CONSTR \| METHOD | DETAIL: FIELD \| CONSTR \| METHOD |  |


---
